# A case of rare pulmonary sequestration complicated with congenital heart disease treated by arterial embolization and atrial defect closure: A case report and review of literature

**DOI:** 10.3389/fcvm.2022.931590

**Published:** 2022-07-22

**Authors:** Mi Tang, Xun Wu, Shijun Hu, Qin Wu, Danni Yang, Chukwuemeka Daniel Iroegbu, Chengming Fan, Jinfu Yang

**Affiliations:** ^1^Department of Cardiovascular Surgery, The Second Xiangya Hospital, Central South University, Changsha, China; ^2^Hunan Agricultural University, Changsha, China

**Keywords:** pulmonary sequestration, atrial septal defect, atrial septal occlusion, interventional therapy, pulmonary vein drainage

## Abstract

Pulmonary sequestration with congenital heart disease is a rare congenital malformation. Herein, we report a 19-month-old toddler diagnosed with right lower pulmonary sequestration, right pulmonary artery dysplasia, right lower pulmonary venous ectopic drainage, and a right-sided heart with an atrial septal defect. The pulmonary sequestration had a rare blood supply, such as confluent arteries with the renal vessels draining into the hepatic veins. Arterial embolization and atrial defect closure were used to treat the rare congenital malformation with satisfactory results.

## Introduction

Pulmonary sequestration, categorized as intralobar or extralobar isolation, is a rare congenital anomaly thought to arise from the accessory lung buds (1). The intralobar lesion’s arterial supply typically arises from the thoracic aorta, while it’s venous drainage via the pulmonary veins. On the other hand, the extralobar lesions drain (venous) via the systemic circulation, including the azygos, hemiazygos, or vena cava (2). Notably, extralobar sequestration, less common than intralobar sequestration, presents early in life with respiratory distress or feeding difficulties, is often associated with other congenital malformations, and accounts for 25% of reported cases.

In pulmonary sequestration, confluent arteries with the renal vessels draining into the hepatic veins are extremely rare. Traditional treatments include surgical resection and supplying artery ligation (3). Notwithstanding, transarterial embolization has since been proposed (4). Some reports using supplying artery embolization to treat pulmonary isolation have reported that the embolic lesions eventually become muscled or even disappeared due to ischemia (5–7).

## Case report

A 19-month-old toddler was admitted to the hospital following a heart murmur discovery. During the fetal period, a routine prenatal color Doppler ultrasound examination at a local hospital detected a congenital cardiac anomaly. However, the examination results were lost by the patient’s family. The patient had recurrent pneumonia after birth. After admission, auscultation revealed a heart murmur and weak breath sounds in the right lung. There was no other notable clinical findings during physical examination and no medical, family, and psychosocial history including genetic information about cardiovascular disease. Cardiac ultrasound examination showed: (i) a dextrorotatory heart; (ii) atrial septal defect (iii) right inferior pulmonary venous drainage; (iv) right pulmonary artery dysplasia; and (v) moderate pulmonary hypertension. The patient was delivered at term with a birth weight of 3.5 kg.

**FIGURE 1:**
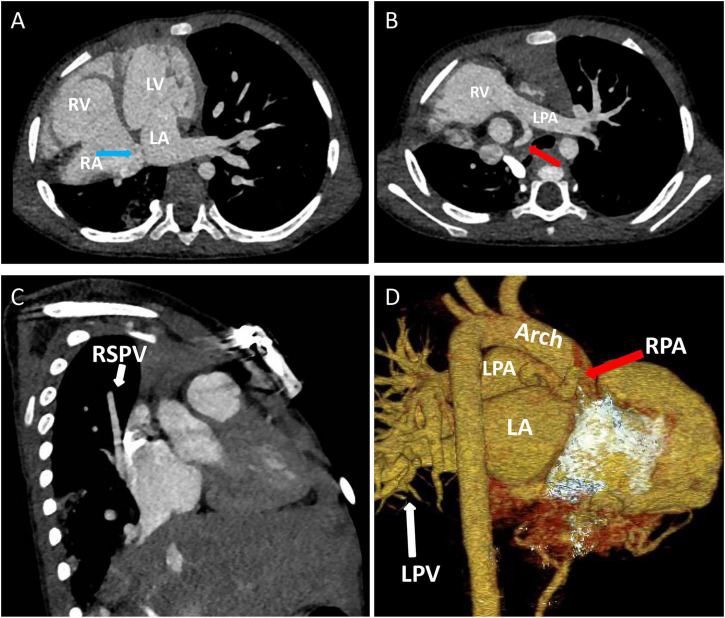
Contrast-enhanced CT of the chest and three-dimensional cardiac computed tomography angiography: **(A)** Atrial septal defect location, ∼9 mm in size (blue arrow). **(B–D)** The right pulmonary artery (red arrow) and right superior pulmonary vein are hypoplastic (white arrow). LA, left atrium; LPA, left pulmonary artery; LPV, left pulmonary vein; LV, left ventricle; RA, right atrium; RPA, right pulmonary artery; RSPV, right superior pulmonary vein; RV, right ventricle.

**FIGURE 2:**
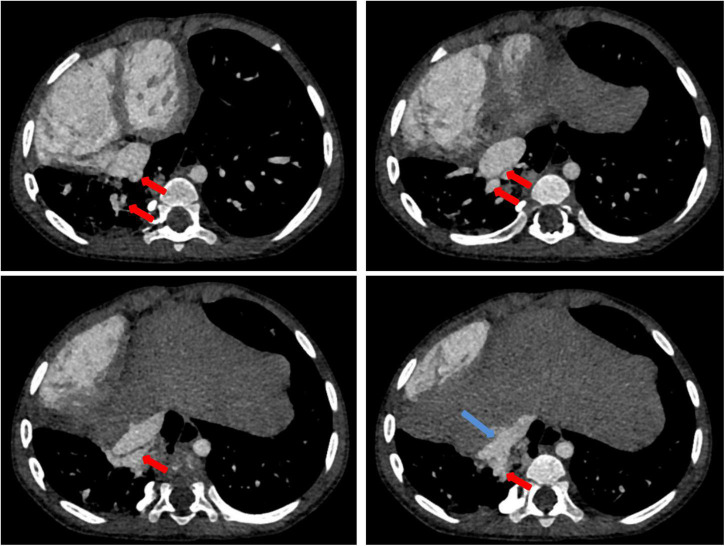
Contrast-enhanced CT of the chest: contrast-enhanced CT shows the isolated pulmonary venous draining (red arrow) into the hepatic vein (blue arrow).

**FIGURE 3:**
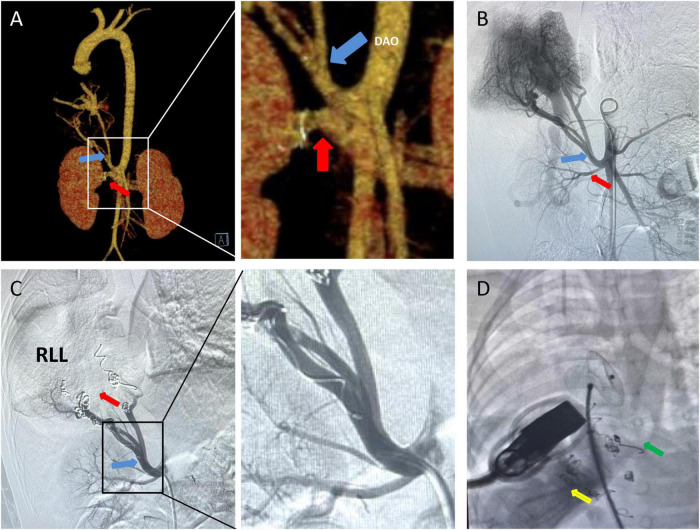
CT 3D reconstruction and Aortic DSA: **(A,B)** A isolates the pulmonary supplying artery (blue arrow) and the renal artery (red arrow) to the abdominal aorta, and isolates the pulmonary supplying artery by approximately 4 mm. **(C)** Nine platinum coils were placed in the isolated pulmonary supplying artery (blue arrow), and no contrast agent entered the distal end of the infarct (red arrow). **(D)** Placement of atrial septal defect occluder (green arrow) under co-guided echocardiography (yellow arrow) and DSA. DAO, decending aorta; RLL, right lower lobe.

Chest X-ray showed a large area of high density in the lower right lung. Chest CT showed a 9 mm defect in the atrial septum (Figure 1A), a significantly small right pulmonary artery (∼5 mm) and a significantly small right superior pulmonary vein (Figures 1B–D), a small and sparsely branched right lung, and the venous of sequestration lung return to the hepatic vein (Figure 2). Considering the patient’s young age and inability to tolerate cardiac and pulmonary surgery, isolated pulmonary embolism and atrial septal occlusion were performed under general anesthesia. Digital subtraction angiography (DSA) and CT 3D reconstruction (Supplementary Video) showed an abnormal blood vessel branching from the right renal artery opening (∼4 mm; Figures 3A–C), and arching toward the right lower lung for blood supply. During the operation, nine coils of equal size were applied to completely block the abnormal blood supply to the right lower lung. Under transthoracic ultrasound guidance, a 14 mm atrial septal occluder was placed through the delivery sheath and ascertained to be adequately fixed, with no residual shunt and intact valvular apparatus (Figure 3D).

The patient recovered well after the operation. Pulmonary infection was controlled with no obvious abnormality following blood examination. Three days postoperatively, echocardiography showed that the occluder was well fixed and there was no residual shunt. The patient had no special discomfort and was discharged 4 days later. No adverse and unanticipated events were indicated during the one-month follow-up period, the patient was strongly advised to come back to the hospital for echocardiography and chest CT reexamination 6 months and 1 year after that.

## Discussion

Pulmonary sequestration refers to the abnormal connection of the lung tissue (part) to the trachea and bronchial tree, with an abnormal vascular supply. Some studies have also suggested that the sequestered lung is a congenital disorder that results from the growth of parapulmonary buds during development. This lung bud is pinched from the caudal foregut, develops its blood supply, and remains independent of the normal tracheobronchial tree (8). However, other authors have suggested that intralobar sequestration can be acquired via bronchial obstruction causing distal infection. It thus stimulates angiogenesis, which utilizes the existing small systemic arteries from the pulmonary ligaments to form a new arterial supply (9).

**FIGURE 4:**
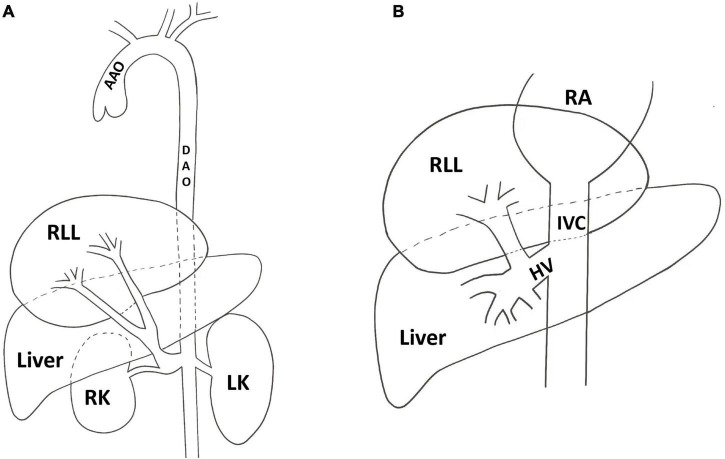
Schematic figure shows the presented case of pulmonary sequestration: **(A)** The arterial supply of the sequestration lung arose from a confluent right renal artery. **(B)** The venous of sequestration lung return to the inferior vena cava via the hepatic vein. AAO, ascending aorta; DAO, decending aorta; HV, hepatic vein; IVC, inferior vena cava; LK, left kidney; RA, right atrium; RK, right kidney; RLL, right lower lobe.

The presented patient was diagnosed with right lower pulmonary sequestration, right pulmonary artery dysplasia, right lower pulmonary venous ectopic drainage, and a right-sided heart with an atrial septal defect. Pulmonary sequestration occurs in 0.15–6.6% of all pulmonary malformations. Two types can be distinguished: intra-leaf (ILS) and extra-leaf (ELS) isolation. With ILS, the abnormal lung tissue is located within the normal lung and visceral pleura, with venous drainage to the pulmonary veins. Its arterial supply is 73% from the thoracic aorta, 20% from the abdominal aorta, and 3.7% from the intercostal arteries. With ELS; however, the mass is located outside the normal lung and within its visceral pleura, with venous drainage to the systemic venous system. Approximately 80% of its arterial supply comes from the thoracic or abdominal aorta, while 15% arises from the subclavian artery, brachiocephalic, spleen, stomach, and intercostal arteries, and 5% from the pulmonary artery (10). In the present case, the arterial supply arose from a confluent right renal artery (Figure 4A), while the venous drainage was to the hepatic vein (Figure 4B), a form of blood supply and drainage that has never been reported.

Extra-leaf (ELS) patients are often accompanied by acute respiratory distress or feeding difficulties after birth. Approximately 60% of children with ELS have congenital malformations, including diaphragmatic hernia, congenital heart disease, elbow syndrome, congenital cyst-adenomatoid malformation (CCAM), or pulmonary dysplasia (11). In the case herein, the patient was born with recurrent episodes of pneumonia and concomitant atrial septal defect.

Traditional treatment is with surgical excision (3). Currently, less invasive endovascular treatments have been reported to prevent complications (12). Platinum coils and polyvinyl alcohol granules are the most frequently used in transcatheter vascular embolization (13). Also, hybrid surgery has been performed for patients with large lung disease and multiple or large feeding arteries or aneurysmal abnormal arteries (14–17). Patients with larger feeding arteries may be at high risk for incomplete embolism, which may require reoperation due to recanalization and symptom recurrence as described in our previous work (18–21). In the present case, the patients feeding artery opening was 4 mm, and the possibility of incomplete embolization was low. Nine platinum coils were placed.

Notably, patients reported having received endovascular therapy recovered better, faster, and had lower complication rates. Given that the abnormal lung parenchyma has not been resected, recurrent hemoptysis and secondary pulmonary infection are the primary complications. Other complications include chest pain and low-grade fever, which might be due to pulmonary infarction after embolization. The patient experienced no postoperative complications, spontaneously recovered after surgery, and was discharged from the hospital 4 days later.

Take-home points: 1. Pulmonary sequestration occurs in 0.15–6.6% of all pulmonary malformations; 2. Pulmonary sequestration can be congenital or acquired via bronchial obstruction causing distal infection; 3. Approximately 80% of its arterial supply comes from the thoracic or abdominal aorta; 4. Transcatheter vascular embolization and hybrid surgery are the most frequently used strategies for the treatment.

## Data availability statement

The original contributions presented in this study are included in the article/Supplementary Material, further inquiries can be directed to the corresponding authors.

## Author contributions

MT, XW, CF, and JY contributed to the conception and design of the study. JY, XW, MT, CI, and CF wrote sections of the manuscript. DY draw the schematic figure. All authors contributed to the manuscript revision, read, and approved the submitted version.

## Conflict of interest

The authors declare that the research was conducted in the absence of any commercial or financial relationships that could be construed as a potential conflict of interest.

## Publisher’s note

All claims expressed in this article are solely those of the authors and do not necessarily represent those of their affiliated organizations, or those of the publisher, the editors and the reviewers. Any product that may be evaluated in this article, or claim that may be made by its manufacturer, is not guaranteed or endorsed by the publisher.

## Supplementary material

Supplementary video Three-dimensional great artery computed tomography angiography and the kidneys.
